# Stability of Near-Surface Nitrogen Vacancy Centers Using Dielectric Surface Passivation

**DOI:** 10.1021/acsphotonics.3c01773

**Published:** 2024-02-14

**Authors:** Ravi Kumar, Saksham Mahajan, Felix Donaldson, Siddharth Dhomkar, Hector J. Lancaster, Curran Kalha, Aysha A. Riaz, Yujiang Zhu, Christopher A. Howard, Anna Regoutz, John J. L. Morton

**Affiliations:** † London Centre for Nanotechnology, UCL, London WC1H 0AH, U.K.; ‡ Department of Electronic & Electrical Engineering, UCL, London WC1E 7JE, U.K.; § Department of Chemistry, UCL, 20 Gordon Street, London WC1H 0AJ, U.K.; ∥ Department of Physics and Astronomy, UCL, London WC1E 6BT, U.K.; ⊥ Department of Physics, IIT Madras, Chennai 600036, India; # Center for Quantum Information, Communication and Computing, IIT Madras, Chennai 600036, India

**Keywords:** diamond, NV centers, quantum sensing, optically detected magnetic resonance, photoluminescence spectroscopy

## Abstract

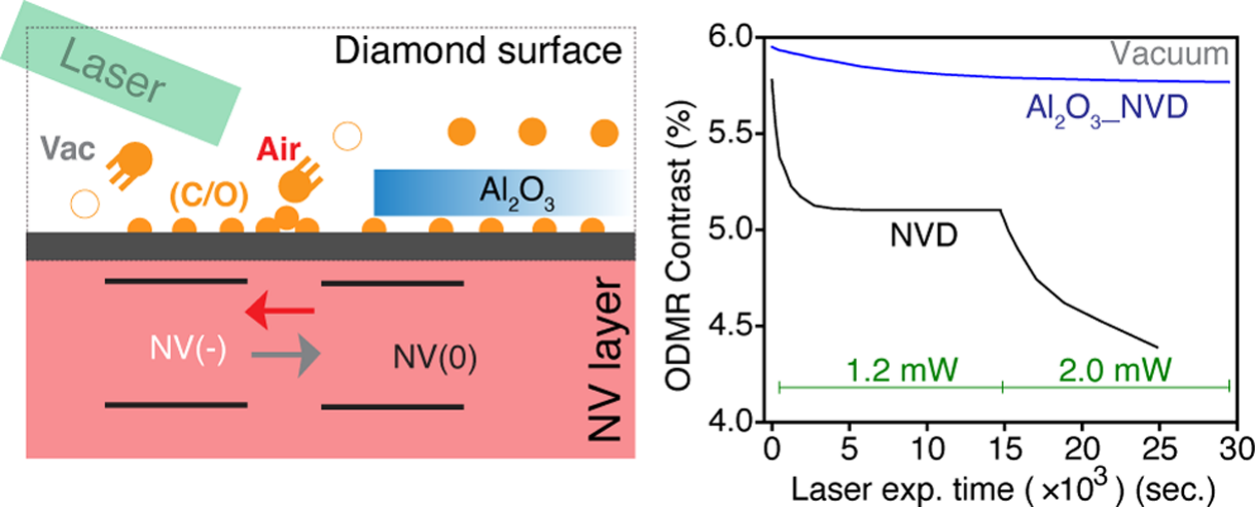

We study the photophysical stability of ensemble near-surface nitrogen vacancy (NV) centers in diamond under vacuum and air. The optically detected magnetic resonance contrast of the NV centers was measured following exposure to laser illumination, showing opposing trends in air compared to vacuum (increasing by up to 9% and dropping by up to 25%, respectively). Characterization using X-ray photoelectron spectroscopy (XPS) suggests a surface reconstruction: In air, atmospheric oxygen adsorption on a surface leads to an increase in NV^–^ fraction, whereas in vacuum, net oxygen desorption increases the NV^0^ fraction. NV charge state switching is confirmed by photoluminescence spectroscopy. Deposition of ∼2 nm alumina (Al_2_O_3_) over the diamond surface was shown to stabilize the NV charge state under illumination in either environment, attributed to a more stable surface electronegativity. The use of an alumina coating on diamond is therefore a promising approach to improve the resilience of NV sensors.

## Introduction

The nitrogen vacancy (NV) center in diamond is an atomistic defect which has emerged as a leading candidate in many solid-state quantum technologies,^1−3^ including quantum sensors to study diverse systems in fields ranging from solid-state physics to complex biological environments.^4−6^ The negative NV charge state (NV^–^) is central to such applications in quantum sensing owing to its optically addressable spin states, long-lived quantum coherence, and room temperature operation.^7^ The ground-state spin-Hamiltonian of NV^–^ center is sensitive to various physical quantities which forms the basis of quantum sensing.^8−10^ The performance of the NV-diamond (NVD) sensor can be determined by measurement sensitivity (; where η_mag._ is AC or DC magnetic sensitivity, *C* is measurement contrast, and *n*_avg._ is the number of NV^–^ photons per measurement)^11^ in addition to the spatial resolution (as low as ∼10 nm)^12^ and operational stability under various environments. Achieving the greatest spatial resolution for quantum sensing requires the positioning of NV centers near the diamond surface (<10 nm); however, the brightness and spin properties of NV centers are compromised near the surface.^2^ For example, the DC magnetic field sensitivity (η_mag._) achieved is ∼17 pT/√Hz for NV centers in the bulk,^13^ which can be compared to ∼1 μT/√Hz for near-surface NV centers.^14^ Furthermore, the stability of near-surface NV centers under nonambient conditions is critical for studying various temperature and pressure-dependent physical phenomenon in solids like magnetic and superconducting materials.^15−17^

The most general diamond surface composition involves nondiamond (sp^2^) carbon, functional groups (C-X*_n_*), dangling bonds, metallic traces, and adsorbed environmental species.^18,19^ Among these, many surface constituents have been identified as a source of local charge traps (e.g., sp^2^ carbon is known to form the double potential well as an electronic trap state)^20^ leading to fluctuating electric and magnetic fields which would degrade the properties of proximal NV centers.^21−23^ NV-diamond quantum sensing protocols typically use high-power nonresonant laser excitation (∼532 nm),^13^ which can lead to significant heating, NV spectral diffusion, ionization of nitrogen atoms (P1 centers), and excitation of various surface constituents.^24−26^ To improve the stability of near-surface NV centers under ambient conditions, numerous atomic functionalization and organic species have been applied on the surface.^20,27−31^ The effects of band bending^32^ and formation of inter-band gap states due to different surface terminations^33,34^ on stability of proximal NVs have been explored using density functional theory (DFT) simulations. Ultrahigh vacuum (UHV) and cryogenic conditions have been reported to degrade the properties of single NV centers in diamond nanopillar structures, while their stability was partially improved upon surface passivation with ultrapure water.^35^ The exact origin of NV degradation near the surface and under different environmental conditions remains unclear and requires attention in order to develop effective mitigation strategies.

In this article, we investigate the instability of near-surface ensemble NV centers under air and vacuum (∼1 × 10^–3^ mbar) at room temperature, measuring NV properties such as optically detected magnetic resonance (ODMR) contrast, as well as characteristic properties of the material surface, following laser illumination. We found the ODMR contrast to vary over the course of (1–2 mW) laser exposure due to NV charge state conversion (NV^–^ ↔ NV^0^) caused by the changing surface chemistry. A stable ODMR contrast and NV charge state were achieved by atomic layer deposition (ALD) of aluminum oxide (Al_2_O_3_, ∼2 nm) on the diamond surface.

## Experimental Details

**Figure 1:**
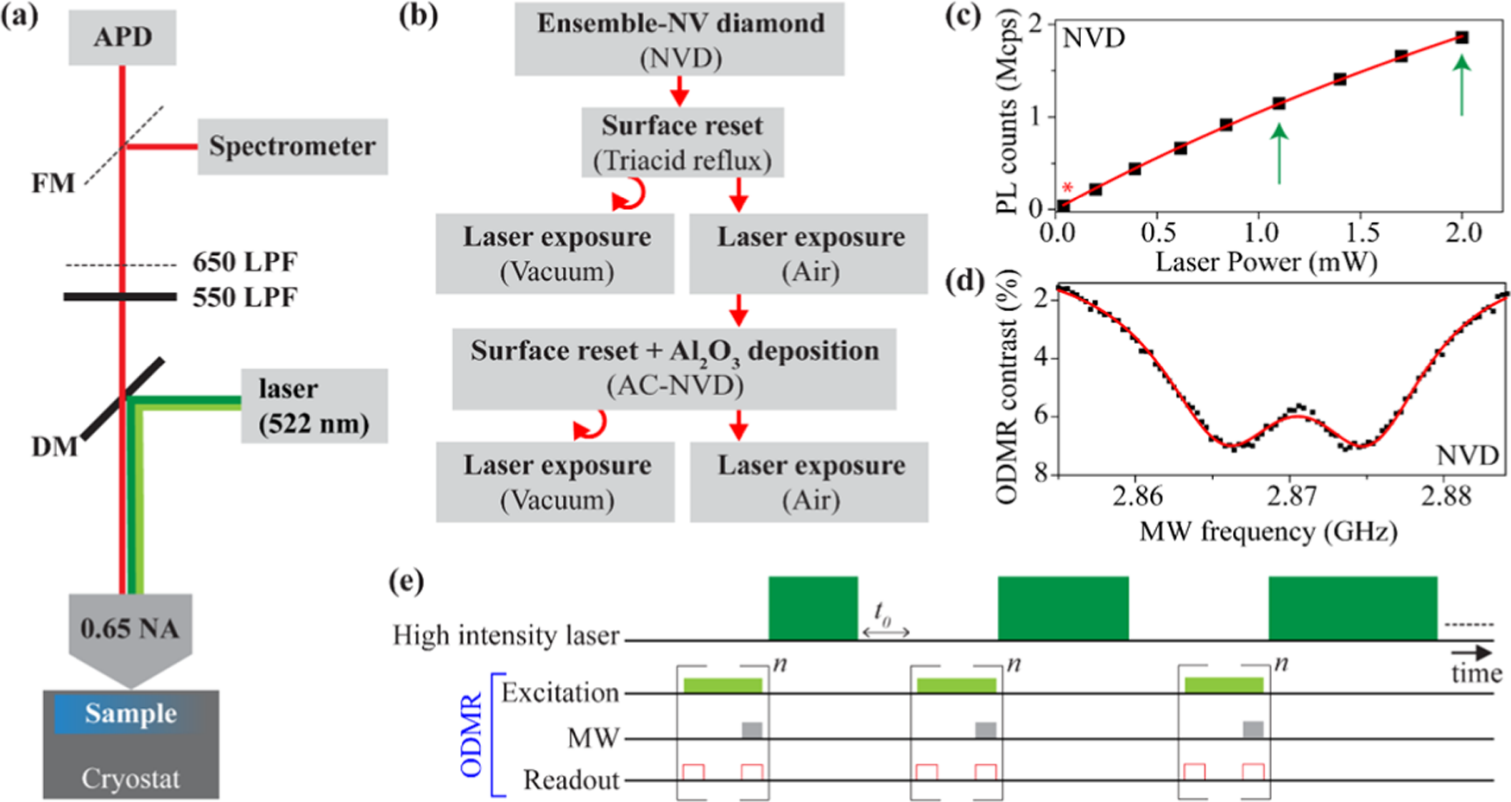
(a) Schematic of the optical measurement setup used for photoluminescence (PL) and ODMR. (b) Summary of the sample preparation and optical measurement steps. (c) Fluorescence saturation measurements (squares) for the NVD sample with a fit (curve) to the saturation curve (see text). Green arrows show the PL signal counts at powers used in subsequent laser exposure experiments. (d) Continuous wave (CW) ODMR spectrum for the NVD sample (black dots) and double Lorentzian fitting (solid red line). (e) Pulse sequence to study ODMR contrast under the influence of periodic high-power laser pulses of increasing duration.

The primary sample investigated here is an electronic grade (100) diamond, ion implanted with nitrogen (^15^N, 3 keV, 1 × 10^13^ ions/cm^2^), supplied by Qnami AG.^36^ The average NV depth was estimated by an average range of N^+^ ions and lattice vacancy profiles using the stopping and range of ions in matter (SRIM) to be ∼5 nm [Figure S1; Supporting Information (SI)].^37,38^ The as-procured sample was acid-refluxed at 255 °C (H_2_SO_4_: HClO_4_: HNO_3_ with 1:1:1 v/v ratio) for 2 h to eliminate nondiamond impurities and increase the oxygen functionalization. We used the acid reflux as a procedure to “reset” the diamond surface in between experiments in different environments. The acid-refluxed sample was termed NV-diamond (“**NVD**”). Sample preparation is summarized in Figure 1b. After the completion of optical measurements on NVD, a ∼2 nm layer of aluminum oxide (Al_2_O_3_) layer was deposited on the sample (NVD → acid reflux →Al_2_O_3_) using a Savannah S200 atomic layer deposition (ALD) system. The thickness of the Al_2_O_3_ layer was found to be ∼2 nm, measured by ellipsometry on a bare silicon substrate (placed together with NVD during ALD deposition). The sample with the deposited Al_2_O_3_ layer is termed alumina-coated NV-diamond (“**AC-NVD**”). Raman and X-ray photoelectron spectroscopy (XPS) spectroscopies were performed using high-purity electronic grade diamond (ELSC20, Thorlabs). As-received ELSC20 diamond plates were acid-refluxed and termed as electronic grade diamond (“**ED**”). A ∼2 nm layer of Al_2_O_3_ was deposited on one ED sample and termed as alumina-coated electronic grade diamond (“**AC-ED**”).

The experimental setup for optical measurements (see Figure 1a) consists of a home-built confocal setup (NA = 0.65) equipped with a Montana Instruments s100 cryostation and 522 nm continuous wave laser (LBX-522; Oxxius). The fluorescence signal was filtered through flip mounted 550 and 650 nm long pass filters (LPFs) and guided toward a single photon counting module (Excelitas Technologies) and photoluminescence (PL) spectrometer (SpectraPro HRS500, Princeton instruments) for ODMR and PL spectroscopy, respectively. For ODMR, the fluorescence signal was collected through a 550 nm LPF in order to observe the maximum effect of NV^0^ emission in ODMR measurements. A laser power of 40 μW was used for ODMR and PL spectroscopy measurements. To study the impact on ODMR contrast from high-power laser illumination, ∼1.2 mW and ∼2 mW laser powers were used. Figure 1c shows the PL intensity as a function of laser power *P*, fitted to the function: , where *I*_sat._ is the saturated intensity (8.5 ± 0.5) Mcps, *P*_sat._ is the saturation power (7.1 ± 0.6) mW, and α denotes the (nonsaturating) background fluorescence. The PL counts shown in Figure 1c as a function of laser power were measured after inserting a neutral density (ND) filter in optical collection path; the actual counts are expected to be ∼10 times higher. For vacuum measurements, the sample chamber was evacuated using a rotary pump (1 × 10^–3^ mbar), while for measurements under ambient conditions, the cryostat head was removed. The experimental scheme for ODMR measurements is shown in Figure 1e. High-power laser illumination was repeatedly applied to the sample with an increasing illumination time after each repetition. The ODMR spectrum was measured using lower powers (*P*_exc._ = 40 μW) after each high-power exposure, following a wait time (*t*_0_) of 60 s. After a cumulative exposure time of ∼1.5 × 10^4^ s at ∼1.2 mW, laser power was increased to ∼2 mW up to a total illumination time of ∼3.0 × 10^4^ s.

In situ Raman spectroscopy was performed using a Renishaw in-Via Raman microscope equipped with a 514.5 nm laser. To estimate the effect of laser illumination, spectral features between ∼1200 and 1900 cm^–1^ were recorded repeatedly under continuous high laser power excitation (2 mW power was applied through an air objective lens of 0.4 NA). For vacuum Raman measurements, the ED and AC-ED samples were placed in a custom-made vacuum compatible glass cell and evacuated to ∼1 × 10^–5^ mbar. The sealed glass cell was then placed under a microscope for spectroscopy. Other Raman measurements were performed under ambient conditions. For XPS sample preparation, the two-dimensional (2D) Raman imaging (area ∼125 μm^2^) of EDs was performed under different environmental conditions. Two laser-exposed samples were prepared under air and vacuum environmental conditions, respectively (Figure S5; SI) for XPS. To eliminate adventitious carbon and adsorbed surface species, the laser-exposed EDs were annealed at 200 °C (2 h) under an argon atmosphere prior to XPS. The XPS was performed using a Thermo Scientific K-Alpha X-ray photoelectron spectrometer with a base pressure of ∼2 × 10^–9^ mbar, equipped with a monochromatic Al K_α_ X-ray source (*h*ν = 1486.7 eV). The X-ray spot size was reduced from the standard 400–100 μm in order to resolve the laser-exposed regions in both samples. The XPS spectra for each sample were recorded at laser-exposed position and another unexposed position (situated at ∼1 mm away from laser-exposed position). The maximum XPS probing depth (*d*_XPS_) at the maximum kinetic energy of 1486.7 eV, i.e., the photon energy of the Al Kα laboratory X-ray source, was estimated by calculating the relativistic inelastic mean free path (IMFP) (*d*_XPS_ = 3 × (IMFP)) using the TPP-2 M model as implemented in the QUASES software package.^39^ The *d*_XPS_ values for the diamond and diamond with Al_2_O_3_ samples were calculated based on C and Al_2_O_3_ models available in the QUASES database and were found to be ∼11.7 and 10.2 nm, respectively. XPS analysis was performed using the Thermo Avantage software package. For the estimation of the relative atomic ratios of carbon and oxygen in different samples, the total peak areas of the C 1s and O 1s core levels and built-in atomic sensitivity factors (ASFs) were used. The change in carbon-to-oxygen atomic ratio (C/O) due to laser exposure was quantified as , where (C/O)_unexp._ and (C/O)_exp._ represent unexposed and laser-exposed positions, respectively. Core level spectra were fitted using the smart background function and Lorentzian–Gaussian sum functions (Figures S6 and S7). The graphitic (sp^2^) and diamond (sp^3^) carbon peak contributions were extracted and the sp^2^/sp^3^ ratio derived. The change in sp^2^/sp^3^ ratio due to laser exposure was quantified as ; where (sp^2^/sp^3^)_exp._ and (sp^2^/sp^3^)_unexp._ represent the laser-exposed and unexposed positions on the sample.

## Results and Discussion

**Figure 2:**
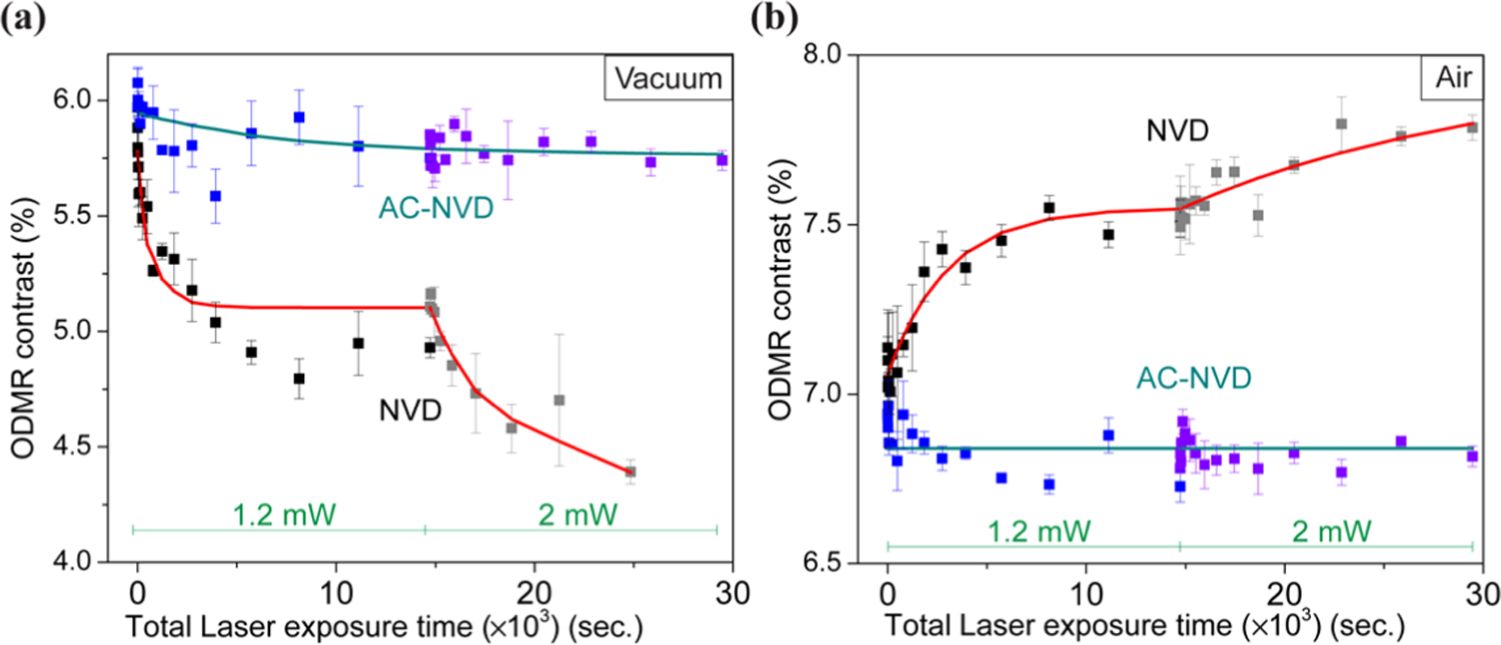
Evolution of ODMR contrast as a function of the total duration of laser power exposure for NVD and AC-NVD samples under (a) vacuum and (b) air environments. A series of laser exposures of increasing duration (from 1 s to 3.6 ks) are first applied using 1.2 mW laser power. After a cumulative exposure time of about 15 ks, the laser power is increased to 2 mW, and the exposure time per data point resets to 1 s time and subsequently increased. The data are fit to exponential decay functions, with separate time constants for the periods of 1.2 and 2 mW laser exposure (see text).

The spin-state-dependent brightness of the NV^–^ center is a fundamental part of its application as a quantum sensor and can be characterized by the ODMR contrast, or the relative change in PL intensity following a change in the spin state.^7^ The ground-state (^3^A) spin triplet (*m*_s_ = 0, ±1) of the defect is characterized by an axial zero field splitting (ZFS) of about 2.87 GHz between spin sublevels *m*_s_ = 0 and ±1. Due to nonaxial lattice strain induced by P1 centers, unoccupied vacancies, implantation-induced lattice structural defects, and local electric field, the degeneracy of spin sublevels *m*_s_ = ±1 is also lifted by a nonaxial ZFS which can vary from 100 kHz to a few MHz.^40−42^ Therefore, in the absence of an external magnetic field, the ODMR spectrum is characterized by a resonance around 2.87 GHz, further split by the nonaxial term, as illustrated by the representative ODMR spectrum for NVD in an air environment shown in Figure 1d. The maximum ODMR contrast for a single NV is about 30%. For ensemble NVs, the ODMR contrast reduces significantly due to several factors such as strain-induced line broadening, interactions with paramagnetic impurities, nontrivial charge dynamics, and inefficient pi-pulse for different NV orientations.^43−48^ The evolution of ODMR contrast under laser illumination under different surface and environmental conditions is shown in Figure 2. For the NVD sample under vacuum, the ODMR contrast exhibits an exponential decay with laser exposure at 1.2 mW and decays further when the laser power increased to 2 mW (Figure 2a), dropping to a contrast of ∼4.5% (or ×0.75 the starting value). The opposite trend is seen when illuminating NVD in air (Figure 2b), where the contrast is seen to rise to ∼7.8% (or ×1.09 the starting value). However, for the AC-NVD sample, the ODMR contrast was found to be relatively independent of high-power laser illumination under either environment, changing by less than ∼0.3%. We fit the time evolution of ODMR contrast under the two consecutive periods of laser exposure at different powers using two exponential functions with a common set of fitting parameters to ensure that the evolution of the contrast is continuous across the two periods. Specifically, we use the functions *y*(*t*) = *C*_0_ + (*C*_1_*e*^–*t*/τ_1_^ + *C*_2_) for *t* < 14,732 s and *y*(*t*) = *C*_0_ + (*C*_1_*e*^–14731/τ_1_^ + *C*_2_*e*^–(*t*–14731)/τ_2_^). Here, τ_1_ and τ_2_ are the decay constants under laser exposure of 1.2 and 2 mW, respectively. *C*_0_ is the ODMR contrast after high laser illumination for an infinite time. The *C*_1_ and *C*_2_ denote the change in the ODMR contrast after laser illumination of 1.2 and 2 mW for infinite time, respectively.

**Figure 3:**
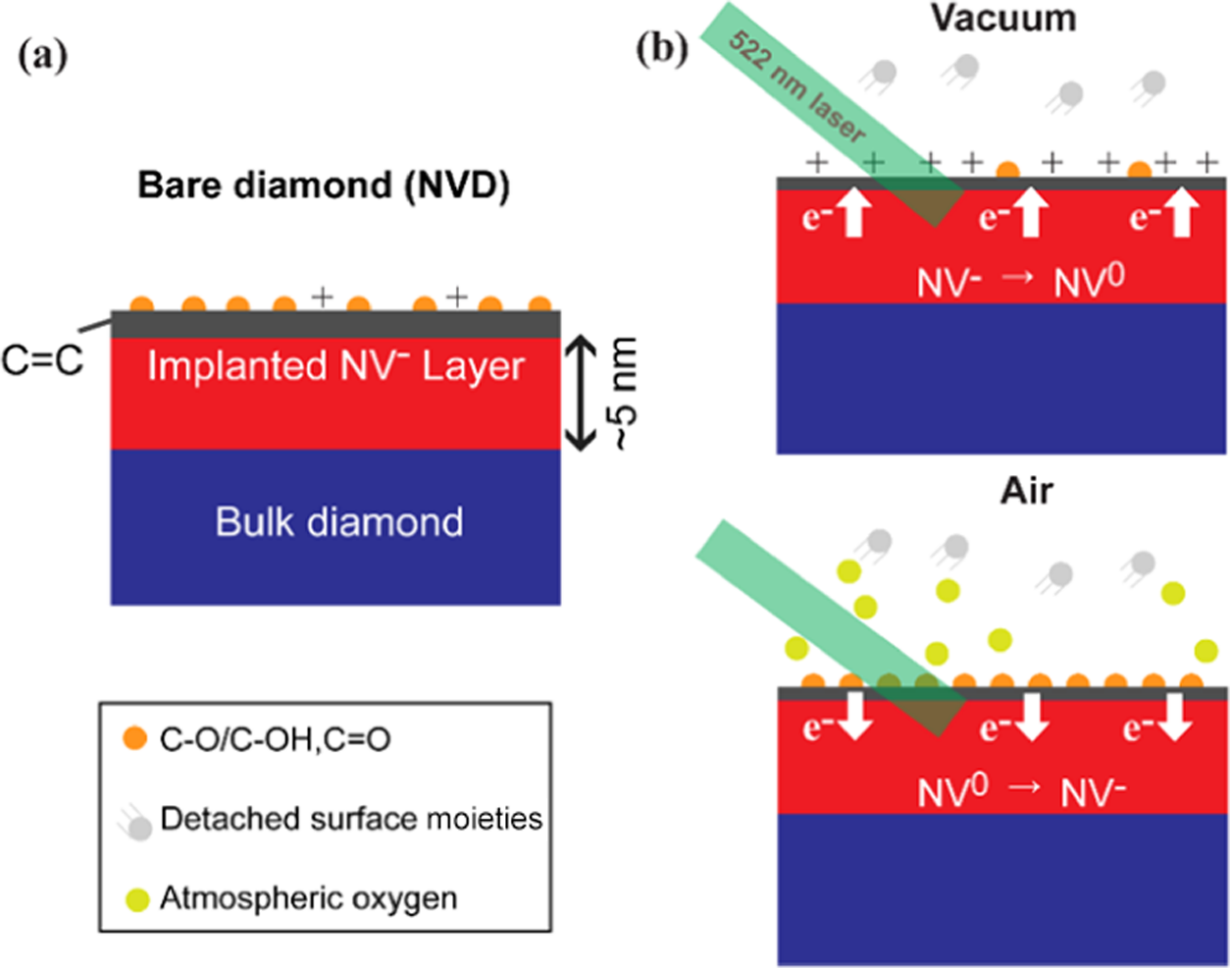
Schematic for proposed mechanism. (a) The acid-refluxed diamond sample (NVD) has a shallow NV-doped layer up to 5 nm from the surface, which consists of nondiamond carbon (gray region) and oxygen functionalities (orange dots) on the surface. (b) Laser exposure under different environments can cause desorption of nondiamond carbon- and oxygen-containing functional groups (in vacuum) or an increase in oxygen termination on the surface (in air). These changes lead to the charge state conversion between NV^–^ and NV^0^.

**Figure 4:**
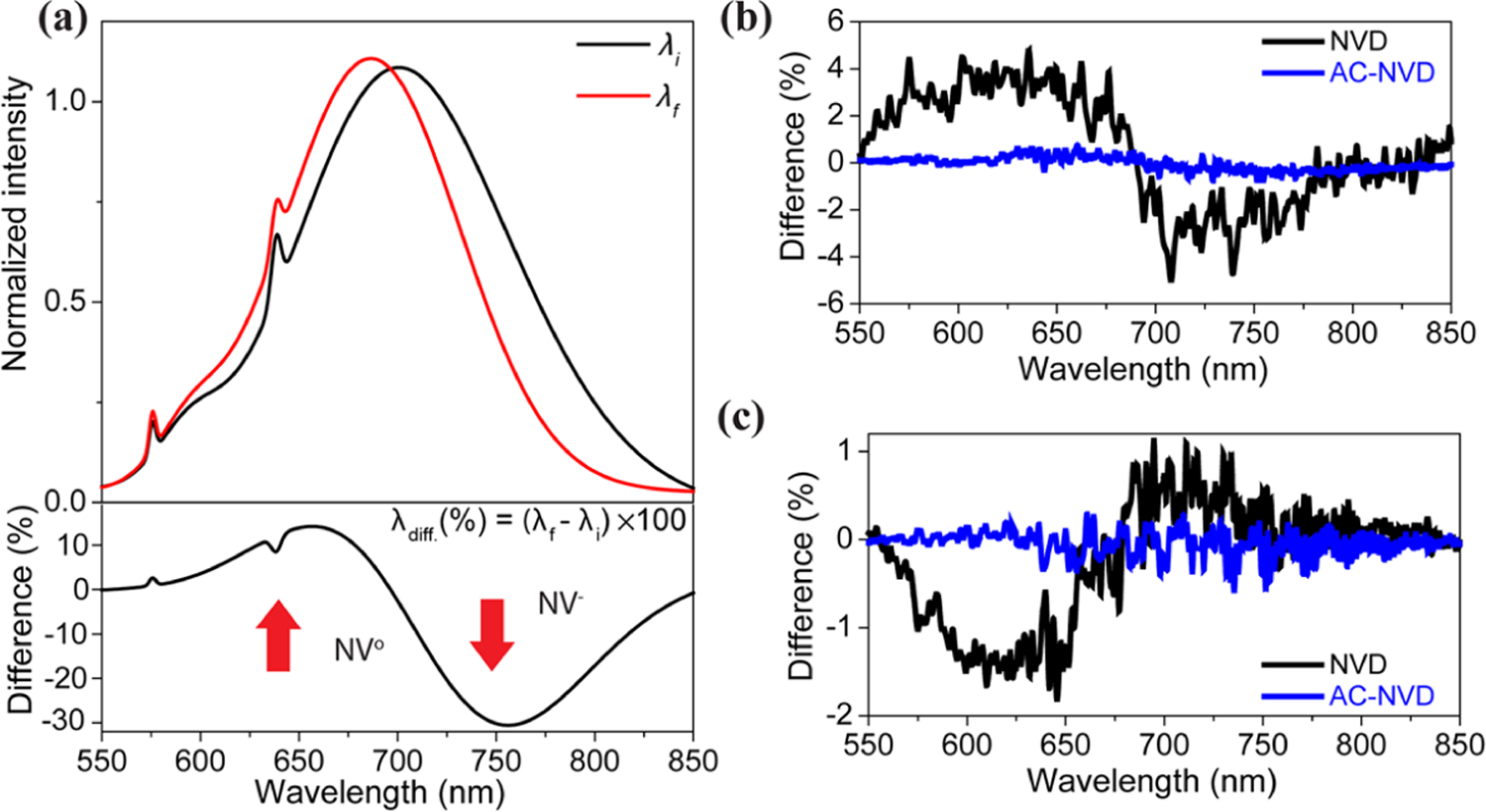
(a) Cartoon representation showing how the PL spectrum evolves as the NV^–^/NV^0^ fraction changes, with the difference shown in the lower panel. Measured difference PL spectra for NVD and AC-NVD are shown in panel (b) under vacuum and panel (c) in air.

**Figure 5:**
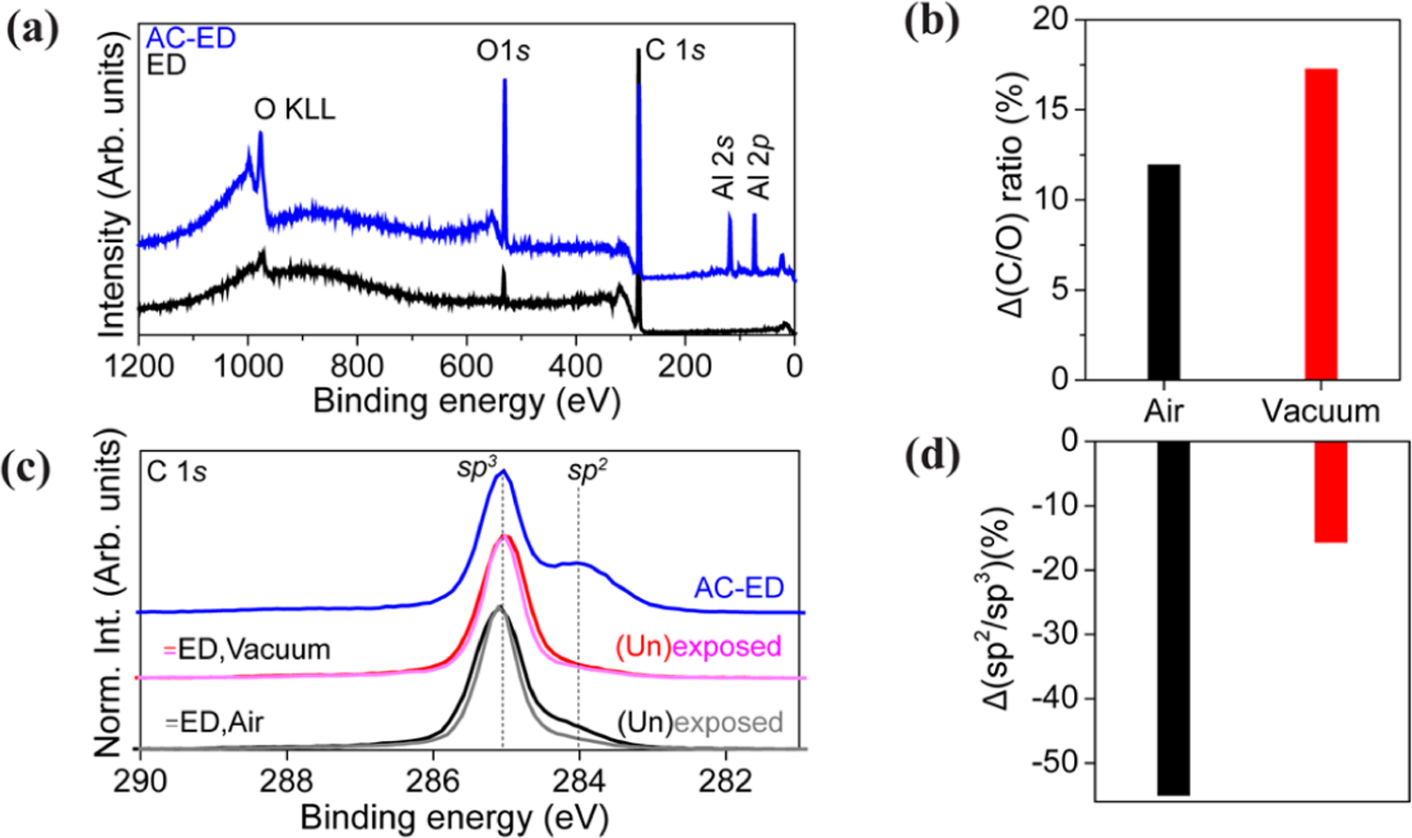
Surface spectroscopy (a) XPS survey spectra of ED and AC-ED samples before laser exposure. (b) Changes in the total carbon-to-oxygen (C/O) ratio of the ED sample as a result of laser exposure, in different environments. (c) C 1s core level spectra showing results from regions in ED exposed to laser (pink, gray), as well as unexposed regions (black, red). The spectra are normalized to the maximum peak height. (d) Changes in the sp^2^/sp^3^ carbon ratio of the ED sample as a result of laser exposure in different environments.

We attribute the observed changes in ODMR contrast to the conversion of NV^–^ to NV^0^, through a mechanism illustrated in Figure 3, supported by measurements described in Figures 4 and 5. Due to near-surface NV fabrication (3 keV N^+^ ions), a high N/NV ratio (∼1%) is present in the NVD sample,^37^ and the residual nitrogen atoms (P1 centers) act as a source of electrons to maintain NV^–^ as preferential NV charge state.^49,50^ The high electronegativity of the oxygen-functionalized surface also helps to maintain their stability.^18^ During laser exposure, excitation of surface species^51^ can result in the detachment of nondiamond carbon and oxygen functionalities and, in the absence of environmental oxygen (e.g., in vacuum), surface electron traps develop which reduce the surface electronegativity, cause upward band bending near the surface, and lead to NV charge state conversion. The reduction in NV^–^ PL emission on top of a background fluorescence signal leads to a gradual reduction in the observed ODMR contrast as a result of this continuously evolving surface composition.

Within an oxygen-rich environment (e.g., in air), laser illumination has the same impact on reducing nondiamond carbon at the surface; however, there is an increase in the oxygen adsorption which increases surface electronegativity (downward band bending) and promotes the NV^–^ charge state (NV^0^ → NV^–^). The evolution of the surface described above (and associated NV charge state conversion) appears to be minimized by the presence of the alumina coating in the AC-NVD sample. Any changes at the diamond surface may be compensated by the alumina providing a stable surface electronegativity on surface.

To further investigate the NV center charge state dynamics, we monitored the PL features of NV^0^ and NV^–^, which are respectively characterized by zero phonon lines (ZPLs) at 575 and 637 nm accompanied by broad sideband emission with maxima around 640 and 700 nm.^8^ Conversion from NV^–^ to NV^0^ leads to a relative increase (decrease) in PL intensity below (above) ∼700 nm, as illustrated in Figure 4a. We recorded PL spectra using ∼40 μW laser power, before and after 1.2 mW laser exposure for 1 h. The difference PL spectra obtained by normalizing and subtracting the spectra before and after high-power laser exposure are shown in Figure 4b,c. Normalized PL spectra under different experimental conditions are shown in Figure S3. The PL spectrum for NVD under vacuum confirms the increase in NV^0^ emission intensity (in the range 550–670 nm) and decrease in NV^–^ intensity (670–800 nm), and the opposite behavior is observed in an air environment. In both cases, negligible changes in the PL spectrum are seen for the AC-NVD sample under vacuum, consistent with NV charge state stability. The comparison of normalized PL of NVD and AC-NVD samples under different environments confirmed that the alumina layer itself does not induce background fluorescence (Figure S4, SI). Overall, these results are consistent with the observed changes in ODMR contrast and the mechanism described in Figure 3.

To gain further insights into the material origin of the observed ODMR contrast changes, XPS was performed (see Figure 5). The surface composition dynamics due to high laser power exposure under different environmental conditions were analyzed by XPS (for details of sample preparation, see Figure S5).^31^ The ED sample exhibits only carbon (C 1s) and oxygen (O 1s) elements within the XPS probing depth (<11.7 nm calculated based on the maximum IMFP). For AC-ED, the Al_2_O_3_ layer was also observed (evidenced by characteristic aluminum peaks (Al 2p and Al 2s) and an intense O 1s peak) in addition to carbon and oxygen (Figure 5a).^52^ The carbon/oxygen relative atomic ratios (C/O) reveal significant surface reconstruction in ED due to laser exposure (Figure S6). The change in the C/O ratio comparing exposed and unexposed positions indicates that laser exposure in a vacuum results in more efficient surface oxygen detachment compared to that in air (Figure 5b). The C 1s core level spectra for different samples were calibrated (peak fitting was used to determine the sp^3^ peak position) to the reported binding energy value for diamond (285.0 eV) and are shown in Figure 5c.^27,53^ The C 1s core level spectra of ED when the laser is exposed in different environments show a variation in spectral shape because of laser exposure (Figure 5c). Peak fitting of the C 1s core level spectra was performed to disentangle, identify, and quantify the different carbon-related chemical states (Figure S6). In ED, nondiamond carbon (sp^2^) is found to be present in addition to diamond (sp^3^) (SI, Figure S6c).^54,55^ The reduction in the ratio of sp^2^ to sp^3^ under laser exposure is much greater in air than in a vacuum (Figure 5d). Peak fitting of the O 1s core level spectra (Figure S7) reveals that the rate of elimination of specific oxygen functionalities (ether/alcohol (C–O–C/C–O–H) or ketone (C=O)) depends on the environment: Due to efficient etching of sp^2^ carbon during laser exposure under air, the concentration of C–O bonds increases, whereas that of C=O bonds decreases. Laser exposure under vacuum induces less efficient sp^2^ carbon etching, and therefore, the rates of removal of C–O and C=O remain similar. In summary, these XPS measurements are consistent with the proposed mechanism (see Figure 3), in which surface oxygen is detached as a result of laser illumination. The C 1s core level spectrum for AC-ED (Figure 5c) reveals a more significant fraction of sp^2^ carbon compared to sp^3^. This can be explained by a change in signal intensity from the diamond sample itself when the ∼2 nm Al_2_O_3_ layer is added on top. This leads to a relative increase in the signal seen from the diamond surface (sp^2^) compared to its bulk (sp^3^). In addition, the O 1s core level spectrum of AC-ED is dominated by Al_2_O_3_, making it difficult to evaluate the diamond surface oxygen functionalization. Due to these factors, we did not perform XPS measurements on the laser-exposed AC-ED sample. However, it will be interesting to explore the AC-ED surface further to see if Al_2_O_3_ alters the diamond functionalization and how the diamond functionality varies under laser exposure. The material changes due to laser exposure were further studied by Raman spectroscopy (Figure S8). Raman spectra of ED and AC-ED samples acquired under low laser excitation power demonstrated the absence of nondiamond carbon and related defects (Figure S8) in addition to a sharp peak at ∼1331.8 cm^–1^ characteristic of diamond. To observe the effects of laser exposure, in situ Raman measurements were performed (see Figure S8 and the SI for details). The diamond-related Raman features remained unchanged for both samples during high laser power exposure (Figure S8). The G band features for ED and AC-ED samples under high-power laser exposure remained inconclusive due to a low signal-to-noise ratio (SNR) in the relevant spectral range (1500–1650 cm^–1^).

## Summary and Conclusions

The instability of near-surface NV centers under nonambient conditions is a long-standing challenge for diamond-based quantum sensing, particularly for work at cryogenic temperatures. To understand the origin of this instability, we combined a study of the optical properties of the NV centers with an analysis of the material composition of the diamond surface. We observe a change of the ODMR contrast for near-surface (∼5 nm deep) NV centers under laser illumination in oxygen-functionalized diamond which we attribute to surface reconstruction under different environmental conditions. In a vacuum, the ODMR contrast was reduced from around 6% to below 4.5%, whereas it increased under air up to over 7.5%. A ∼2 nm layer of Al_2_O_3_ was deposited on the diamond surface which successfully led to a stable ODMR contrast, even under laser exposure. The origin behind these changes in the ODMR contrast was revealed by PL and XPS spectroscopies to arise from NV charge state switching caused by surface dynamics. In vacuum, owing to lack of atmospheric gases, electron traps develop on the surface and the NV^–^ charge state is converted into NV^0^. Atmospheric oxygen inhibits the development of such traps and increases the NV^–^ charge state fraction. The Al_2_O_3_ layer prohibits both the degradation of the surface as well as adsorption of environmental oxygen, achieving a more stable NV charge state. The Al_2_O_3_–oxygen–diamond surface is shown to be resilient against optical excitation in vacuum but requires further investigation under low temperature conditions, while the NV spin coherence properties should be analyzed in such materials to assess its potential for quantum sensing. The use of alumina coating could be extended to help stabilize single near-surface NV centers in planar and nanopillar diamond structures. A single NV-diamond AFM probe with stability under different environmental conditions might be achievable using such optimized passivation of the surface.

## Supporting Information Available

Additional experimental details, SRIM simulation of 3 keV ^15^N^+^ implantation in diamond, ODMR, PL, XPS and Raman spectroscopy results (PDF)
