# Supplementary material for: Stability of Near-Surface Nitrogen Vacancy Centers Using Dielectric Surface Passivation
Source: ACS Photonics. 2024 Feb 14;11(3):1244–51. doi: 10.1021/acsphotonics.3c01773 (PMC10958592; doi:10.1021/acsphotonics.3c01773)
Supplement: Supplementary file 1 — ph3c01773_si_001.pdf [file ph3c01773_si_001.pdf]

Supporting information

Stability of near surface nitrogen vacancy centers using dielectric surface passivation

Ravi Kumar[1,,‡], Saksham Mahajan[2,‡], Felix Donaldson[1], Siddharth Dhomkar[1,5,6], Hector J. Lancaster[4], Curran Kalha[3], Aysha A. Riaz[3], Yujiang Zhu[3], Christopher A. Howard[4], Anna Regoutz[3] and John J.L. Morton[1,2]*

[1]London Centre for Nanotechnology, UCL, London WC1H 0AH, UK

[2]Department of Electronic & Electrical Engineering, UCL, London WC1E 7JE, UK

[3]Department of Chemistry, UCL, 20 Gordon Street, London WC1H 0AJ, UK

[4]Department of Physics and Astronomy, UCL, London WC1E 6BT, UK

[5]Department of Physics, IIT Madras, Chennai 600036, India

[6]Center for Quantum Information, Communication and Computing, IIT Madras, Chennai 600036, India

*Email: ucanrku@ucl.ac.uk

‡These authors contributed equally

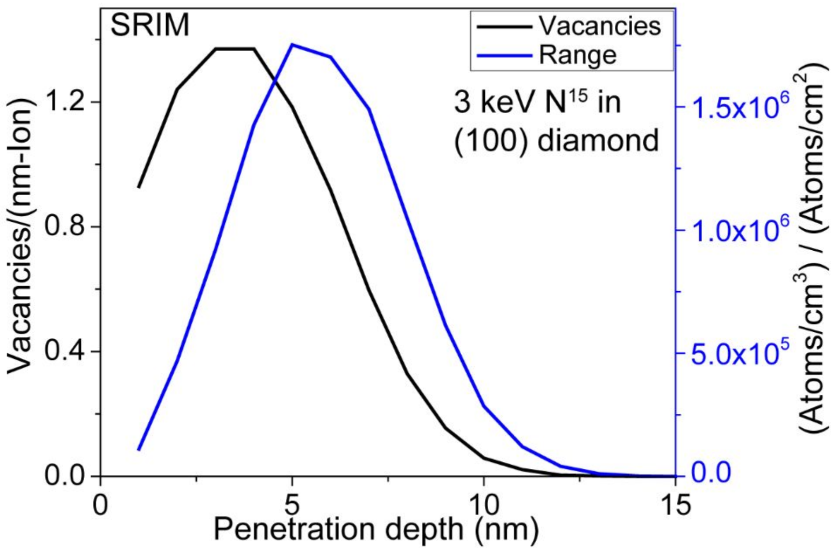

Figure S1. SRIM simulation of 3 keV ^{15}N implantation in (100) diamond. The threshold energy (T_d) for displacement of carbon atoms from lattice position was taken to be 37.5 eV.[1] The average depth of lattice vacancies was ~ 3.5 nm (maximum depth of NV ~ 12 nm). Whereas, the average penetration depth was ~ 5.5 nm (maximum depth of implanted Nitrogen ~ 15 nm). Considering that nitrogen ions remain immobile during annealing procedure (post implantation NV fabrication step), the NV concentration profile should closely follow nitrogen stopping range curve (Blue).

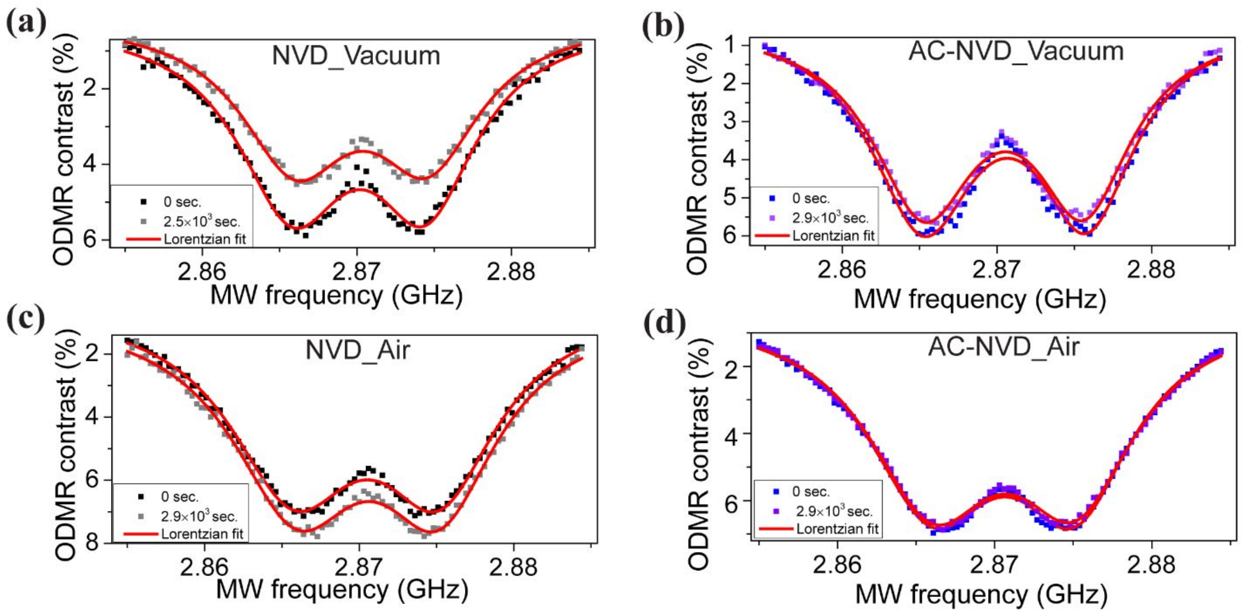

Figure S2. ODMR spectra before and after laser exposure for (a) NVD under vacuum (b) AC-NVD under vacuum (c) NVD under air and (d) AC-NVD under air respectively.

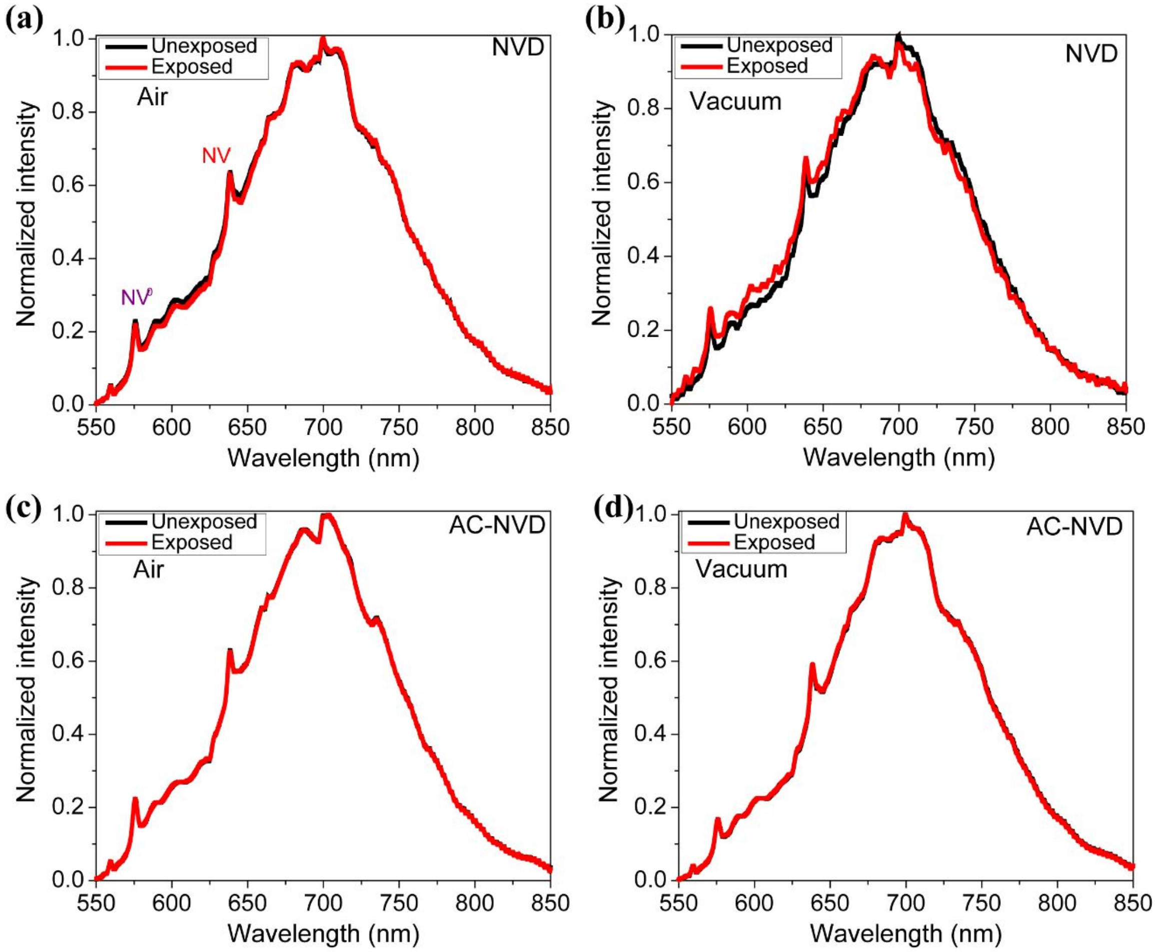

Figure S3. Normalized PL spectra under different conditions before and after high power laser exposure. (a) and (b) show normalized spectra for BD sample under air and vacuum respectively. (c) and (d) show the PL spectra of AC-ED sample under air and vacuum respectively.

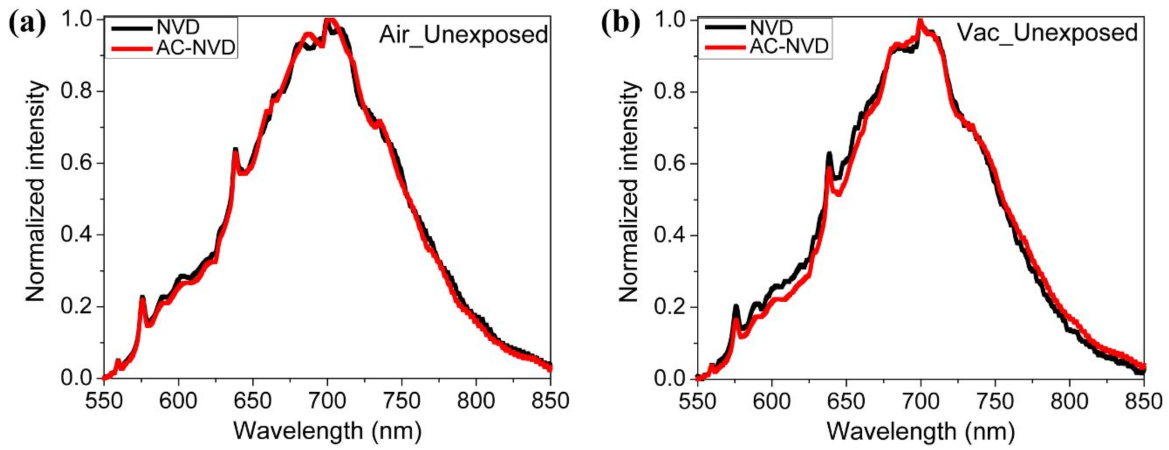

Figure S4. Normalized PL spectra of NVD and AC-NVD samples before high power laser exposure under (a) air and (b) vacuum respectively.

The sample preparation for the XPS has been summarized in fig. S5. Fig. S6(a) shows the Al *2p* core level spectrum for AC-ED. Fig. S6(b) shows the calculated C/O atomic ratios for ED samples laser exposed under different atmospheric environments. For the quantification of different carbon species, the C 1*s* core level spectra were fitted using Gaussian Lorentzian sum functions. A representative fitted C 1*s* core level spectrum for ED_Air Exposed (Laser exposed under air environment) is shown in Fig. S6(C). For ED sample at laser unexposed and exposed positions under air environment (ED_Air), the binding energy positions for sp^3 carbon were found to be 285.3 eV and 285.0 eV, respectively. For ED sample at laser unexposed and exposed positions under vacuum environment (ED-Vac.), the binding energy positions for sp^3 carbon were found to be 285.2 eV and 284.7 eV, respectively. The sp^2 carbon for each sample is shifted ~ -1.0 eV w.r.t. sp^3 carbon positions. The calculated sp^2/sp^3 ratio for different samples has been shown in fig. S6(d).

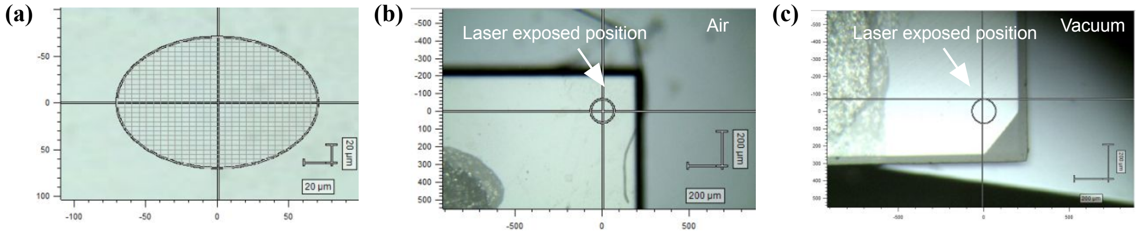

Figure S5. Sample preparation for XPS measurements. (a) shows the Raman mapping pattern over diamond. (b) and (c) show the laser exposed region of different samples under air and vacuum respectively.

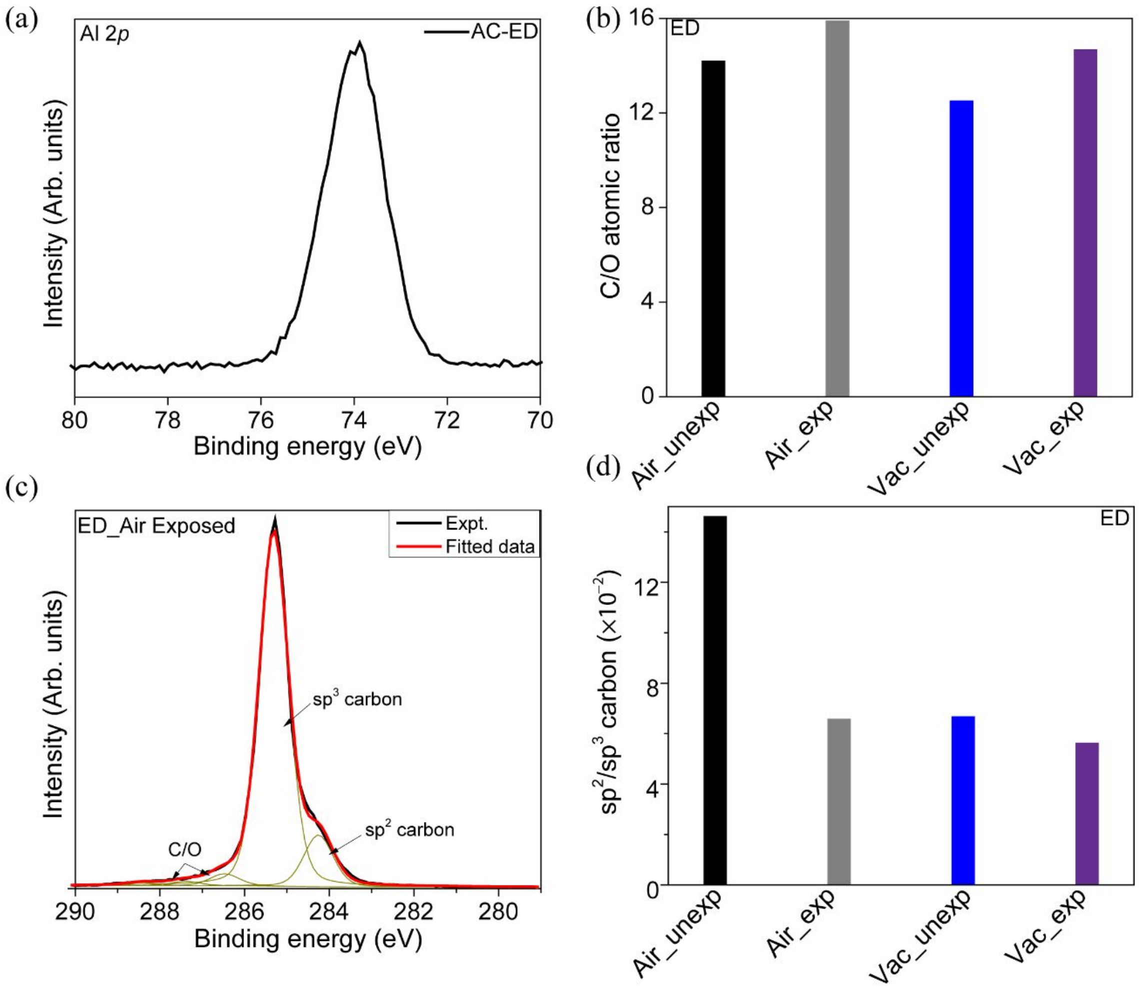

Figure S6. (a) Al 2*p* core level spectrum for AC-ED sample. (b) C/O atomic ratio for EDs after high power laser exposure under different environments. (c) Representative C 1*s* fitted core level spectrum for sample ED after laser exposure under air environment. (d) The sp^2/sp^3 carbon ratio for different ED samples estimated from peak fit analysis of the corresponding C 1*s* core level spectra.

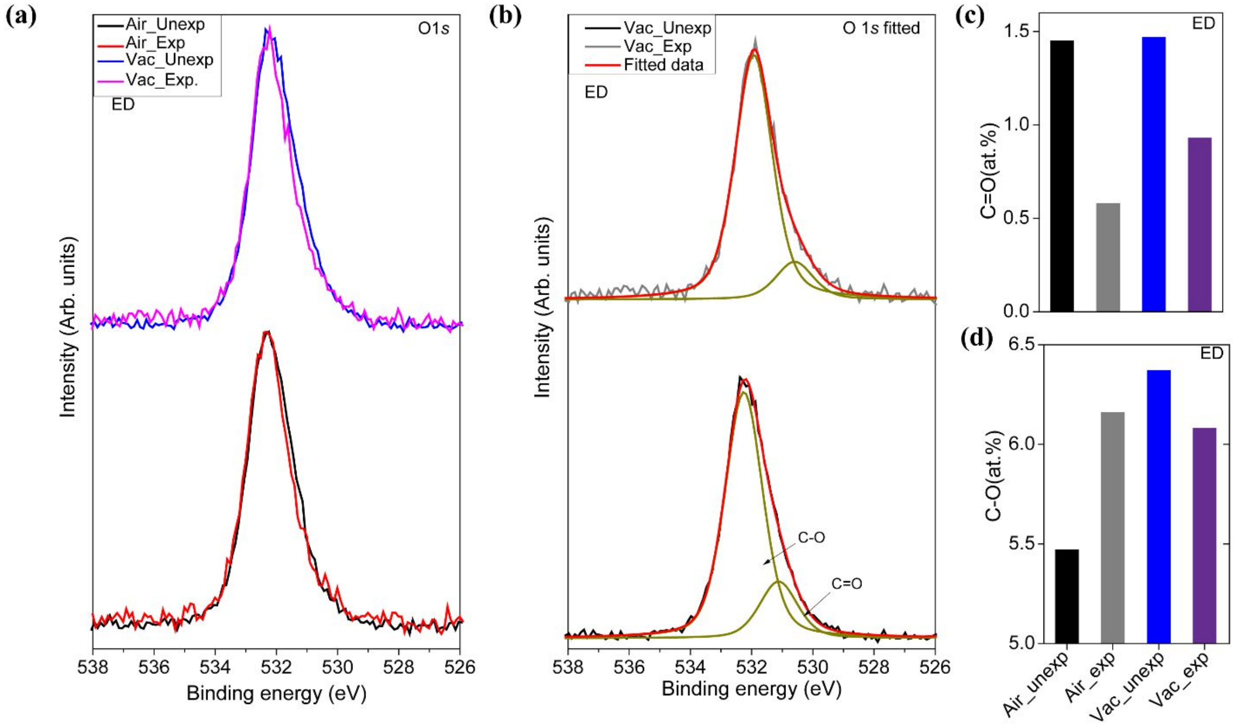

Figure S7. (a) O 1*s* core level spectra for EDs after laser exposure under different environments. (b) Peak fit of the O 1*s* core spectrum for ED sample after laser exposure under vacuum environment. (c) and (d) show C-O (%) and C=O (%) respectively for laser exposed EDs under different environments. Here, C-O represents ether (C-O-C) or alcohol (C-O-H) and C=O represents ketone surface functionality.[2,3]

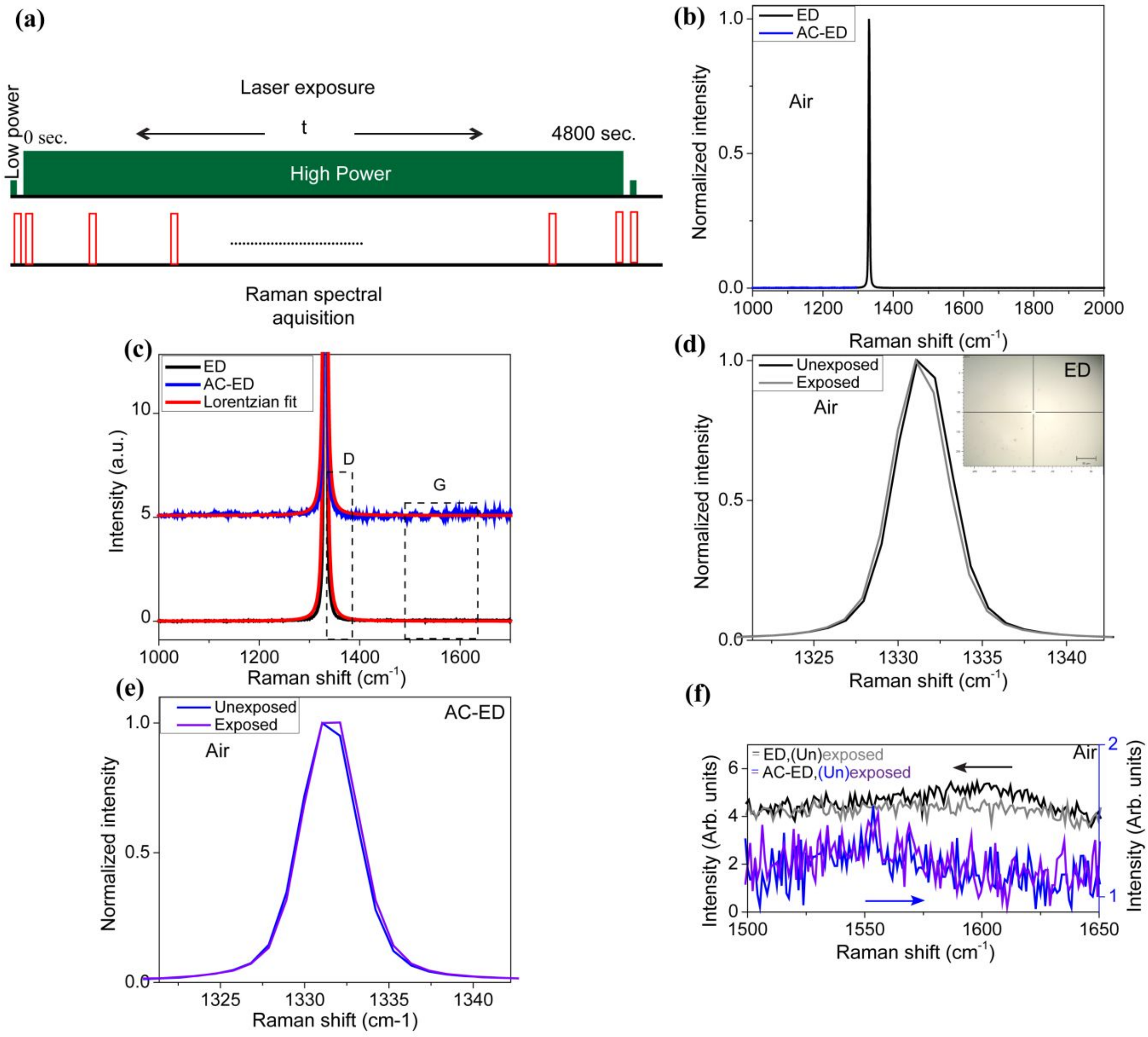

Figure S8. a) Experimental scheme for Raman spectroscopy. (b) Raman spectra for ED and AC-ED samples at low excitation laser power and corresponding zoomed D and G band regions. There is no observable feature related with defects (D band) and graphitic carbon (G band) at low laser excitation power. (d) and (e) show the comparison of characteristic diamond Raman features (spectra were acquired at low laser power) before and after high laser power exposure under air environments for ED and AC-ED samples respectively. (f) Normalized Raman spectra for ED and AC-ED samples under high power laser illumination in air.

References:

1. Koike, J.; Parkin, D.; Mitchell, T., Displacement threshold energy for type IIa diamond. *Appl. Phys. Lett.* **1992,** *60*, 1450-1452.

2. Baldwin, C.; Downes, J.; McMahon, C.; Bradac, C.; Mildren, R., Nanostructuring and oxidation of diamond by two-photon ultraviolet surface excitation: An XPS and NEXAFS study. *Phys. Rev. B* **2014,** *89*, 195422.

3. Sangtawesin, S.; Dwyer, B. L.; Srinivasan, S.; Allred, J. J.; Rodgers, L. V.; De Greve, K.; Stacey, A.; Dontschuk, N.; O'Donnell, K. M.; Hu, D., Origins of diamond surface noise probed by correlating single-spin measurements with surface spectroscopy. *Phys. Rev. X* **2019,** *9*, 031052.
